# Geographical association of biodiversity with cancer and cardiovascular mortality rates: analysis of 39 distinct conditions

**DOI:** 10.3389/fpubh.2024.1368017

**Published:** 2024-04-30

**Authors:** Qiaochu Xu, Bingjie Qu, Li Li, Ying Chen

**Affiliations:** ^1^Department of Geography and Planning, School of Environmental Science, University of Liverpool, Liverpool, United Kingdom; ^2^Wisdom Lake Academy of Pharmacy, Xi’an Jiaotong-Liverpool University, Suzhou, China; ^3^Department of Health and Environmental Sciences, Xi’an Jiaotong-Liverpool University, Suzhou, China

**Keywords:** biodiversity, richness of birds, cause-specific mortality, cancer, cardiovascular disease, ecological study, epidemiology

## Abstract

**Background:**

Biodiversity has been recognized as a positive contributor to human health and wellbeing. Cardiovascular disease and cancer are the two most significant global health burdens, and understanding their relationship with biodiversity forms an essential step toward promoting biodiversity conservation and human health.

**Methods:**

The species richness of birds is a common indicator of biodiversity, given their vast numbers, distinctive distribution, and acute sensitivity to environmental disturbances. This ecological study utilized avian observation data derived from the eBird database, human health data from the International Health Metrics and Evaluation, and county-level statistics, including population characteristics, socio-economics, healthcare service, residential environment, and geographic and climatic characteristics in 2014. We aimed to extensively explore the individual associations between biodiversity (i.e., avian species richness) and age-standardized cause-specific mortalities for different types of cancers (29 conditions) and cardiovascular diseases (10 conditions) across the United States (US).

**Results:**

Our multiple regression analyses that adjusted for a variety of socio-demographic and geographical factors showed that increased rarefied species richness of birds was associated with reduced mortality rates for three of the five most common cancers, namely, tracheal, bronchus, and lung cancer, breast cancer (in women only), and colon and rectal cancer. For cardiovascular conditions, a similar relationship was observed for ischemic heart disease and cerebrovascular disease—the two most frequent causes of mortality. This study provided extended details regarding the beneficial effects of biodiversity on human health.

## Introduction

A healthy ecosystem provides multiple services that may boost human health (1). In maintaining ecosystem function, stability, and resilience, biodiversity is an indispensable factor (2). One proposed theory suggested that exposure to, and experience of, biodiversity can be linked to human health under four crucial categories: harm reduction, capacity restoration, capacity building, and harm initiation (3). An increasing body of literature has been providing evidence that biodiversity is positively associated with human mental health and wellbeing (4). However, the relationship between biodiversity and physical health remains a topic of scholarly debate. Our prior research identified a significant association between higher levels of species diversity and improved health outcomes, including extended life expectancy (5). However, to understand the mechanism underlying the observed relationship between biodiversity and the mortality rate of certain causes, further investigations into specific types of conditions are required.

Neoplasm and cardiovascular issues constitute major parts of the global health burden and are listed as the top two causes of premature death (6). Globally, cancers of the tracheal, bronchus, and lung predominated as principal causes of cancer-related mortality, followed by colon and rectum cancer and stomach cancer (7). From 1980 to 2014, despite declining mortality rates, cardiovascular diseases remained the leading cause of death in the US, with significant disparities persisting across counties in mortality rates from ischemic heart disease and stroke (8).

Bird species richness is among the most commonly employed indicators of biodiversity and has proven to be an effective measure of environmental health in regional-scale studies (9, 10). Compared to other taxa commonly utilized for representing biodiversity, such as herbaceous plants, trees, insects, and small mammals, birds offer several distinct advantages (10–12). Birds are plentiful, spanning both terrestrial and wetland habitats, with significant spatial variability (13–15). They also display a heightened sensitivity toward environmental disruptions, attributed to their unique ecological niches from which they gather cumulative feedback from other species; thereby, the numerical trends among avian species often mirror those among other species (16). Importantly, compared to the concept of greenness, the presence of birds is relatively independent of socio-economic factors as an indicator of naturalness, especially in urban areas.

In this study, we thoroughly investigated the spatial associations between species richness of birds and cause-specific mortalities for 29 different cancer types and 10 distinct cardiovascular conditions. We tested the following hypothesis that, across the US, we can detect the association between the species richness of birds and mortality rates of different cancers and cardiovascular diseases. Our findings provide additional epidemiological knowledge regarding the beneficial effect of biodiversity on human health.

## Materials and methods

Our research presented an ecological study conducted across the US with health and biodiversity data sampled at the county level (or their equivalents). We collected two groups of data: (1) rarefied species richness of birds as a predictor variable for biodiversity, and (2) age-standardized mortality rates for various types of cancers and cardiovascular diseases as outcome variables.

The county-level bird species list was obtained from the eBird database,^1^ the details of which can be found in references (5, 17). Data were obtained on 1st July 2022, which included all submissions to the dataset up to that point. Our research enrolled bird data gathered throughout the US between 2013 and 2015, encompassing over six million events and approximately a hundred million recorded observations across 3,137 counties. After excluding counties with inadequate observations (50 events or fewer), we employed a rarefaction method to ensure comparable levels of bird species richness across each county by adjusting for variations in sampling efforts (5, 18). We undertook repetitive random sampling of the bird population in each county, with the sample size (907) based on the county with minimal bird abundance. This approach aimed to achieve optimal rarefaction accuracy under the condition of maximum samples (19). This particular process was conducted using R (version 4.3.1) and the package “vegan” package, utilizing its internal “rarefy” function (20). We incorporated bird data from 2013 to 2015 because it aligned with the health measurement data from 2014, which was the most current health data accessible during our study. In the current study, for the study year 2014, a total of 2,751 county-level data on rarefied species richness of birds were collected, validated, and computed for the subsequent investigations.

Annual mortality rates of specific cancers and cardiovascular diseases were collected from the Institute for Health Metrics and Evaluation (21). Adhering to the framework of the Global Burden of Disease (GBD) study, cancers and cardiovascular diseases were further divided into 10 and 29 distinct diagnoses, respectively (7). These subcategories are shown in Table 1. Data from 2014, as the most recent accessible data, were used in this study.

**Table 1:** County-level statistics of the US.

Variables		Number (%), or median (IQR), or mean (SD)
Biodiversity		
Rarefied species richness of birds		99.4 (85.2, 111.6)
Population characteristics		
Population size		31,118 (13,040, 79,614)
Sex, male		49.6% (49.0, 50.5%)
Ethnicity, white alone		91.9% (81.0, 95.8%)
Age, years		
0–9		12.1% (10.9, 13.3%)
10–19		12.9% (11.9, 13.9%)
20–29		11.8% (10.4, 13.5%)
30–39		11.5% (10.5, 12.5%)
40–49		12.2% (11.3, 13.2%)
50–59		14.7% (13.7, 15.6%)
60–69		12.0% (10.7, 13.4%)
70–79		7.2% (6.0, 8.4%)
80 and over		4.3% (3.5, 5.2%)
Socio-economics		
Education level, 25 years and over		
Less than a high school diploma		11.7% (8.6, 16.3%)
A high school diploma only		33.9% (29.2, 38.8%)
Completing some college or associate’s degree		30.8% (27.5, 34.3%)
A bachelor’s degree or higher		20.0% (15.6, 26.8%)
Median household income (annual, US dollar)		46,046 (39,999, 53,304)
Gross domestic product *per capita* (annual, US dollar)		6.0% (4.7, 7.4%)
Unemployment rate, age < 65		15.5% (12.0, 19.9%)
Poverty rate		36,880 (27,536, 49,631)
Healthcare service		
Health insurance coverage, age < 65		86.1% (82.4, 89.7%)
Physicians per 1,000 population		0.9 (0.4, 1.6)
Residential environment (Rural–Urban Continuum Area Code)		
1. (Metro areas, 1 million population or more)		412 (14.9)
2. (Metro areas, 250 thousand to 1 million population)		366 (13.3)
3. (Metro areas, population fewer than 250 thousand)		333 (12.1)
4. (Urban population of 20 thousand or more, adjacent to a metro area)		209 (7.6)
5. (Urban population of 20 thousand or more, not adjacent to a metro area)		89 (3.2)
6. (Urban population of 2,500 to 19,999, adjacent to a metro area)		514 (18.7)
7. (Urban population of 2,500 to 19,999, not adjacent to a metro area)		361 (13.1)
8. (Completely rural or less than 2,500 urban population, adjacent to a metro area)		166 (6.0)
9. (Completely rural or less than 2,500 urban population, not adjacent to a metro area)		301 (10.9)
Geographic and climatic characteristics		
Longitude		−90.3 (−98.6, −83.1)
Latitude		38.5 (34.7, 41.9)
Average temperature, Fahrenheit scale		53.1 (46.5, 59.7)
Annual precipitation, inches		39.2 (26.8, 46.8)
Cause of death	(Ranking)	
Neoplasms	–	202.91 (30.10)
Tracheal, bronchus, and lung cancer	(1)	61.14 (16.94)
Breast cancer (women only)	(2)	26.11 (3.87)
Prostate cancer (men only)	(3)	25.88 (4.75)
Colon and rectum cancer	(4)	24.37 (4.19)
Pancreatic cancer	(5)	12.8 (1.34)
Leukemia	(6)	9.48 (0.92)
Ovarian cancer (women only)	(7)	8.61 (0.97)
Non-Hodgkin lymphoma	(8)	8.58 (0.94)
Liver cancer	(9)	6.35 (1.67)
Esophageal cancer	(10)	5.48 (0.96)
Kidney cancer	(11)	5.14 (0.76)
Bladder cancer	(12)	5.10 (0.69)
Brain and nervous system cancer	(13)	5.09 (0.63)
Stomach cancer	(14)	4.43 (1.18)
Multiple myeloma	(15)	3.97 (0.48)
Cervical cancer (women only)	(16)	3.77 (1.03)
Uterine cancer (women only)	(17)	3.72 (0.65)
Malignant skin melanoma	(18)	3.37 (0.64)
Lip and oral cavity cancer	(19)	1.93 (0.47)
Larynx cancer	(20)	1.36 (0.38)
Gallbladder and biliary tract cancer	(21)	1.15 (0.22)
Non-melanoma skin cancer	(22)	1.07 (0.22)
Other pharynx cancer	(23)	1.01 (0.27)
Mesothelioma	(24)	0.99 (0.36)
Thyroid cancer	(25)	0.55 (0.06)
Nasopharynx cancer	(26)	0.29 (0.14)
Testicular cancer (men only)	(27)	0.28 (0.06)
Hodgkin lymphoma	(28)	0.39 (0.05)
Other neoplasms	–	6.17 (0.63)
Cardiovascular diseases	–	273.22 (55.61)
Ischemic heart disease	(1)	169.59 (44.65)
Cerebrovascular disease	(2)	52.87 (11.15)
Hypertensive heart disease	(3)	9.95 (7.37)
Cardiomyopathy and myocarditis	(4)	7.93 (3.07)
Atrial fibrillation and flutter	(5)	7.58 (1.74)
Aortic aneurysm	(6)	4.29 (0.63)
Rheumatic heart disease	(7)	3.38 (0.79)
Peripheral vascular disease	(8)	2.67 (0.7)
Endocarditis	(9)	2.59 (0.59)
Other cardiovascular and circulatory diseases	–	12.38 (2.34)

Causes-specific mortality rate, reported as “mean (SD)” per 100,000 population. Ranking, based on the average mortality rates, counted separately for cardiovascular disease and neoplasm, starting from the most frequent cause of death. SD, standard deviation. CIs, Confidence intervals.

In addition, information on population characteristics (size, gender, age, and ethnicity), socio-economics (educational level, median household income, gross domestic product *per capita*, and rates of unemployment and poverty), healthcare service (coverage of medical insurance and number of physicians per residential population), residential environment (the Rural–Urban Continuum Code), and geographical and climatic characteristics (temperature, precipitation, latitude, and longitude) were also collected as covariates at the county level for confounding effect adjustment.

Multiple regression analyses were applied to examine the association of rarefied species richness of birds with each mortality rate caused by distinct conditions, with adjustment for potential confounding factors. The average temperature was not included in the final models due to the violation of multi-collinearity with longitude. For the studied 2,751 counties, we mapped county-level bird species richness with covariates and each disease mortality rate. The cardiovascular diseases and cancers significantly associated with the bird species richness were further identified through multivariate linear regressions. We reported a regression coefficient for rarefied species richness of birds against each assessed disease mortality, which measures the size of the effect for each cause-specific mortality rate per unit on the change of rarefied bird species. Meanwhile, a broader confidence interval signifies a greater magnitude of uncertainty. All statistical analyses were performed in R (version 4.3.1). A *p*-value <0.001, two-tailed, was considered as statistical significance, aiming for conservative results with a low likelihood of false positive findings.

## Results

**Figure 1:**
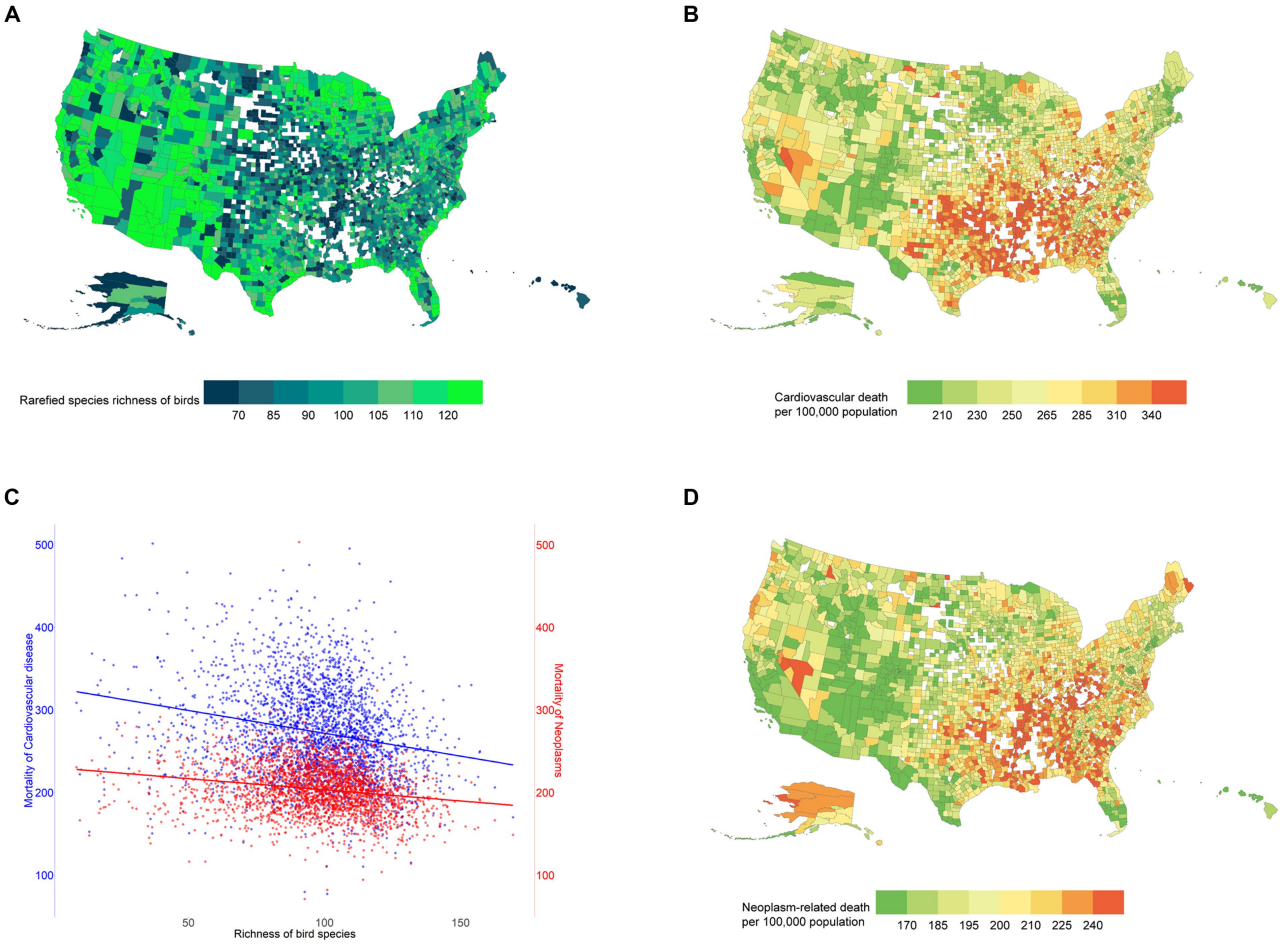
The geographical distributions of the rarefied species richness of birds and mortality rate of cardiovascular disease and neoplasm across the US in 2014. **(A)** Rarefied species richness of birds. **(B)** Mortality rates of cardiovascular disease. **(C)** Scatter plots between both mortality rates of cardiovascular disease and neoplasm and rarefied species richness of birds. **(D)** Mortality rates of neoplasm.

The average rarefied species richness of birds at the county level was 97.0 (standard deviation (SD) 22.0). The rate of mortality caused by each of 29 specific cancer types and 10 distinct cardiovascular conditions is also reported, as well as descriptive statistics on studied covariates, in Table 1. Unspecified analysis suggested, in general, that there was a geographical correlation between rarefied species richness of birds and cancer and cardiovascular mortalities (Figure 1). Specified by distinct types of diseases, the univariate regression analysis suggested a significant association between the rarefied species richness of birds and 19 out of 29 types of cancer. Meanwhile, a substantial link existed between the rarefied species richness of birds and 7 out of 10 cardiovascular ailments without further adjustments.

**Figure 2:**
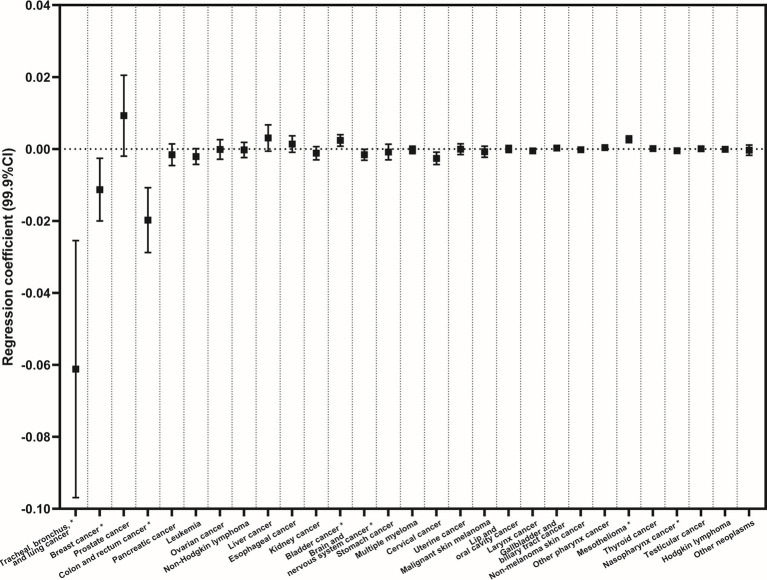
Regression analyses for the associations between different types of neoplasms and rarefied species richness of birds after adjustments for potential confounding factors. (Adjusted confounding factors: population size, gender, age, ethnicity, educational level, median household income, gross domestic product *per capita*, rates of unemployment, poverty rates, coverage of medical insurance, number of physicians per residential population, the Rural–Urban Continuum Code, temperature, precipitation, and latitude).

After adjusting for study covariates, statistically significant negative associations were observed, with considerable effect sizes. These associations included rates of mortality caused by tracheal, bronchus, and lung cancer, as indicated by the regression coefficient (99.9% confidence interval): (−0.061 (−0.097, −0.025)), breast cancer (in women only, −0.011 (−0.020, −0.003)), and colon and rectal cancer (−0.020 (−0.029, −0.011)) (Figure 2). Associations were also found with bladder cancer (0.002 (0.001, 0.004)), brain and nervous system cancer (−0.002 (−0.003, −0.000)), cervical cancer (in women only, −0.003 (−0.004, −0.001)), mesothelioma (0.003 (0.002, 0.004)), and nasopharynx cancer (−0.0005 (−0.0008, −0.0002)); however, the mortality rates and effect sizes of associations were much smaller (Figure 2).

**Figure 3:**
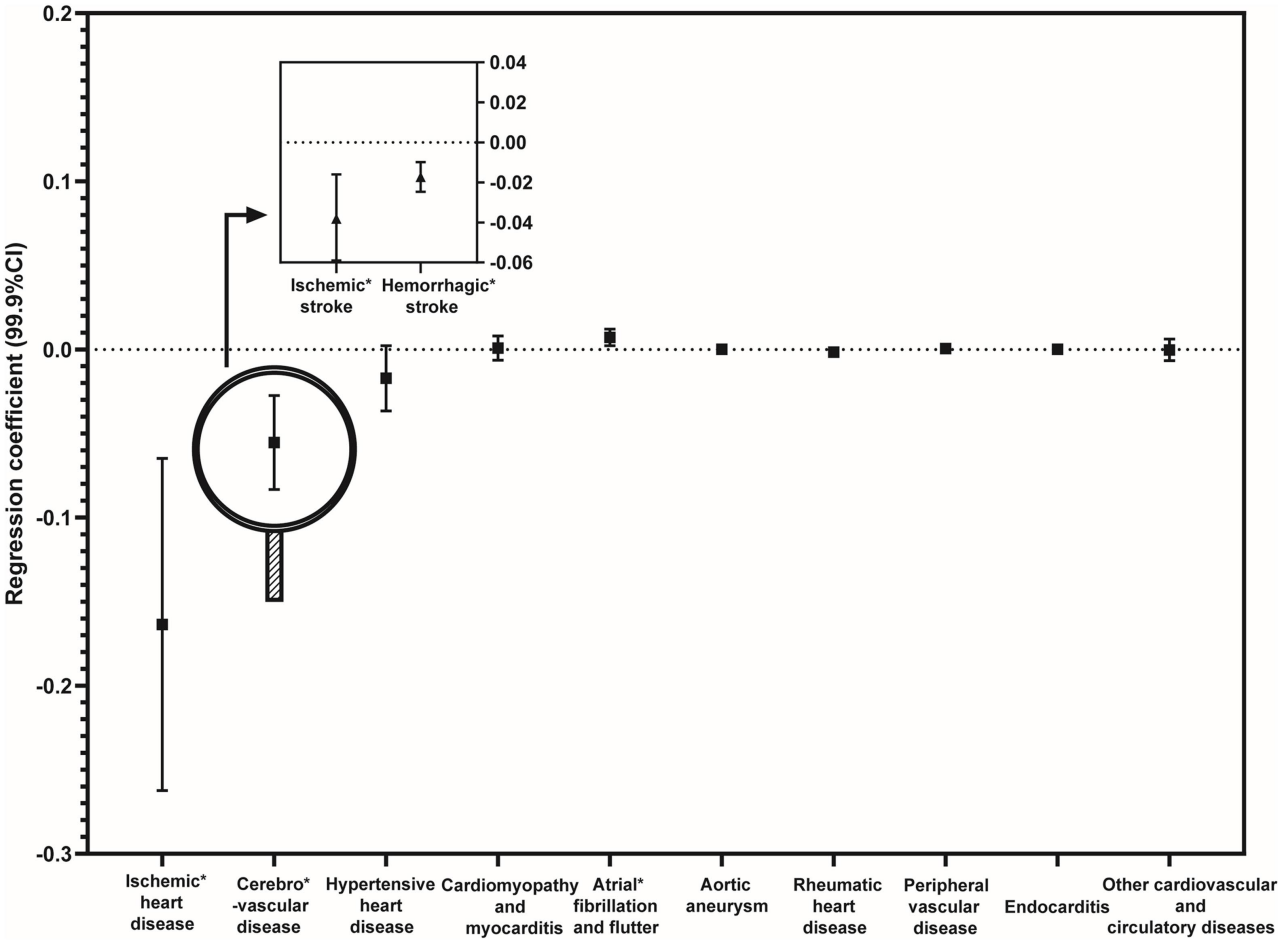
Regression analyses for the associations between multiple specific cardiovascular diseases and rarefied species richness of birds after adjustments for potential confounding factors. (Adjusted confounding factors: population size, gender, age, ethnicity, educational level, median household income, gross domestic product *per capita*, rates of unemployment, poverty rates, coverage of medical insurance, number of physicians per residential population, the Rural–Urban Continuum Code, temperature, precipitation, and latitude).

Repeated multivariate analyses on the rates of cardiovascular mortalities revealed three significant associations, with considerable effect sizes observed in mortalities from ischemic heart disease, as indicated by the regression coefficient (99.9% confidence interval): [−0.164 (−0.262, −0.065)] and cerebrovascular disease [−0055 (−0.083, −0.027)] (Figure 3).

## Discussion

Our previous research demonstrated that, in general, the species richness of birds was negatively associated with the mortality rates of cancer and cardiovascular diseases, indicating a synergistic pattern between biodiversity and the physical health of the human population (5). By analyzing the GBD categorization of individual types of cancers and cardiovascular diseases, it was shown that the interactive relationship was largely attributed to biodiversity’s associations with reduced mortality rates of tracheal, bronchus, and lung cancer, breast cancer (in women only), colon and rectal cancer, and ischemic heart disease and cerebrovascular disease (including ischemic stroke and hemorrhagic stroke), within the cancer and cardiovascular disease categories, respectively. These conditions are considered among the most concerning burdens of human health problems. For example, lung cancer is the most frequent cancer in men, whereas breast cancer is the most common in women. The combined mortality rate of ischemic heart disease and cerebrovascular disease accounts for over 80% of the total cardiovascular-related deaths (8).

The One Health approach takes a holistic perspective, recognizing the interconnection between human health, the natural environment, and biodiversity. This holistic thinking also suggests that mitigating threats to biodiversity can concurrently alleviate stressors on human health and vice versa. Together with our results, increased species richness of birds was found to be associated with reduced mortality rates of tracheal, bronchus, and lung cancer, non-cancer chronic respiratory diseases, and cardiovascular diseases. The results highlight the potential common threats underlying the interactive relationship between biodiversity and physical health. According to current literature, these threats, which influence both avian diversity and the incidence of breast, colon, and rectal cancers, might include ambient air pollution, environmental chemicals, artificial night light, and nutrition deficiency (5, 22–27).

Biodiversity, however, may also have its own functional role in promoting human health. For example, studies within residential environments have shown that exposure to areas with high biodiversity, measured by the area of greenspace, is associated with reduced incidences of cancer and respiratory and cardiovascular diseases (1, 28, 29). In particular, a study conducted in Spain suggested that increased greenness was associated with a decreased risk of breast cancer (30). Furthermore, there is often a reported “luxury effect” of biodiversity, where wealthier neighborhoods tend to have a higher level of biodiversity (31). This raises a crucial question about the equitable distribution of biodiversity’s benefits as a public good for human health. Although our approach does not establish a direct cause-and-effect relationship between biodiversity and health outcomes, there is a well-established theoretical basis indicating that this association carries significance (3). This conceptual framework identifies multiple biodiversities–human health pathways, including positive interactions such as the provision of medicines and food, reducing exposure to air and noise pollution, attention restoration, stress recovery, and encouraging physical activity, as well as negative interactions such as the increasing risk of allergies and pathogens. Particularly, exposure to environmental pollution is one of the potential drivers that link biodiversity with human health. The current understanding of the biological mechanisms behind cancer indicates that it arises from a combination of environmental and genetic factors (32). A variety of external influences can contribute to the development of human cancers; for instance, air pollution serves as a significant hazard factor for respiratory infections, cardiac disease, lung cancer, and breast cancer (23, 32). Many common environmental chemicals and air pollutants are carcinogens that specifically target breast tissue, instigate relevant hormonal pathways, or amplify the susceptibility of the breast tissue to carcinogenesis (23). Air pollutants, especially particulate matter (PM), could potentially facilitate inflammatory alterations in the microenvironment of lung tissue, allowing pre-existing mutated cell populations to proliferate (33). For ischemic heart disease, air pollution leads to an increased incidence of coronary artery disease. Long-term exposure to certain concentrations of PM2.5 could increase the risk of stroke and death due to cerebrovascular disease (34). Air pollution-induced oxidative stress mechanisms are responsible for cardiovascular and cerebral damage and trigger subsequent inflammation and gene activation (35). In our study, birds, as a proxy of biodiversity, are often sensitive to environmental changes in ways that reflect the overall quality of the environment in a specific region, including aspects such as air and water quality, as well as ecosystem stability (16, 36). In fact, there is also relevant pictorial evidence showing a similar spatial distribution pattern between air pollution, indicated by PM2.5, and bird diversity across our study areas in the US (37). Beyond the common threats to both biodiversity and human health, accessibility to biodiversity and nature could also explain the observed association in our findings. Ample evidence suggests that interacting with the natural environment and proximity to greenness can reduce stress and encourage physical activity (38–40). These correlations become stronger with the degree of proximity and duration of exposure to such green spaces (41, 42). In terms of stress recovery theory, physiologically measured stress signals recovered faster and more completely when participants were exposed to natural rather than built-up environments (43). The potential mechanism for the physiological findings proposed that parasympathetic nerves have a prominent response in the face of nature and that their primary function was to restore and maintain the body’s energy resources (44). In patients with coronary heart disease, acute psychological stress has been validated to induce transient myocardial ischemia, while long-term pressure could potentially augment the hazard of recurrence and mortality from ischemic heart disease (45). These findings are consistent with the results inferred from our study. When specific cancer types were discussed, there was a significant association between depression and breast cancer mortality (46, 47). Meanwhile, a meta-analysis incorporating 157 prospective studies revealed a significant association between stress-related psychosocial factors and poorer survival outcomes for lung and breast cancer patients (48). In summary, the mechanisms through which biodiversity interacts with human health are likely multifaceted. Further research is needed to clarify the direct cause–effect relationship between biodiversity and specific health components or to uncover the common driving factors behind the observed associations between biodiversity and health.

Overall, this is a large national-scale study analyzed at the county level. We made a significant effort to collect and adjust for many variables in the following aspects: population characteristics, socio-economics, healthcare service, residential environment, and geographic and climatic characteristics to minimize the potential confounding effects. The study utilized direct health indicators of disease mortality rather than other health measurements (e.g., prevalence or incidence) due to possible variations in disease recording standards across the US and the scarcity of unbiased data. Certain health measurements can be influenced by detection abilities and survival bias. However, on the other hand, this research is limited by its study design, which is an ecological study. The primary concern that cannot be ignored is the uncertainty surrounding the correlation between exposure and outcome within individual cases. In addition, the potential bias resulting from population movements could affect data collection. It is currently unknown whether a significant trend of mass migration exists for reasons other than those considered in this study. Rather than conducting a mediation effect analysis on a specific covariate, we addressed all covariates as potential confounding factors for the adjustment, thereby precluding us from discerning whether there exist any corresponding mediators between the exposure and the outcome. This study provides novel insights into the interactive relationship between biodiversity and human health, as well as the possible mechanisms underlying biodiversity’s health-benefiting effect. Yet, those mechanisms are far from clear and are contested.

## Data availability statement

The original contributions presented in the study are included in the article/supplementary material, further inquiries can be directed to the corresponding author.

## Ethics statement

Ethical approval was not required for the study involving humans in accordance with the local legislation and institutional requirements. Written informed consent to participate in this study was not required from the participants or the participants’ legal guardians/next of kin in accordance with the national legislation and the institutional requirements.

## Author contributions

QX: Writing – original draft, Visualization, Software, Methodology, Formal analysis. BQ: Writing – review & editing, Formal analysis, Data curation. LL: Writing – review & editing, Supervision, Conceptualization. YC: Funding acquisition, Writing – original draft, Supervision, Conceptualization.

## Footnotes

^1^ https://ebird.org
